# Auditory hallucinations activate language and verbal short-term memory, but not auditory, brain regions

**DOI:** 10.1038/s41598-021-98269-1

**Published:** 2021-09-23

**Authors:** Paola Fuentes-Claramonte, Joan Soler-Vidal, Pilar Salgado-Pineda, María Ángeles García-León, Nuria Ramiro, Aniol Santo-Angles, María Llanos Torres, Josep Tristany, Amalia Guerrero-Pedraza, Josep Munuera, Salvador Sarró, Raymond Salvador, Wolfram Hinzen, Peter J. McKenna, Edith Pomarol-Clotet

**Affiliations:** 1 FIDMAG Hermanas Hospitalarias Research Foundation, C/. Dr. Antoni Pujadas 38, 08830 Sant Boi de Llobregat, Barcelona Spain; 2 CIBERSAM, Madrid, Spain; 3 Universitat de Barcelona, Barcelona, Spain; 4 Benito Menni Complex Asistencial en Salut Mental, Sant Boi de Llobregat, Spain; 5 Hospital de Sant Rafael, Barcelona, Spain; 6 Hospital Mare de Déu de la Mercè, Barcelona, Spain; 7 Hospital Sagrat Cor de Martorell, Barcelona, Spain; 8 Hospital de Sant Joan de Déu, Barcelona, Spain; 9 ICREA (Institució Catalana de Recerca i Estudis Avançats), Barcelona, Spain; 10 Universitat Pompeu Fabra, Barcelona, Spain

**Keywords:** Schizophrenia, Neural circuits

## Abstract

Auditory verbal hallucinations (AVH, ‘hearing voices’) are an important symptom of schizophrenia but their biological basis is not well understood. One longstanding approach proposes that they are perceptual in nature, specifically that they reflect spontaneous abnormal neuronal activity in the auditory cortex, perhaps with additional ‘top down’ cognitive influences. Functional imaging studies employing the symptom capture technique—where activity when patients experience AVH is compared to times when they do not—have had mixed findings as to whether the auditory cortex is activated. Here, using a novel variant of the symptom capture technique, we show that the experience of AVH does not induce auditory cortex activation, even while real speech does, something that effectively rules out all theories that propose a perceptual component to AVH. Instead, we find that the experience of AVH activates language regions and/or regions that are engaged during verbal short-term memory.

## Introduction

Auditory verbal hallucinations (AVH) are a major symptom of schizophrenia, estimated to occur in around 70% of patients^1^. While the clinical features of the phenomenon are well established—AVH may be single or multiple, are often but not always derogatory, and may be experienced inside or outside the head (or both)^2^−the mechanism or mechanisms underlying them remain obscure.

Theoretical approaches to AVH include so-called ‘cognitive’ models, which argue that they are a manifestation of non-perceptual processes, for example inner speech that fails to be labeled as internally generated^3^, or memories whose vividness and/or intrusiveness leads them to be misinterpreted as perceptions^4,5^. The other main approach, the ‘neurological’ or ‘perceptual’ model, proposes that AVH are in some sense genuinely perceptual in nature. In its simplest form this approach dates back to the beginning of the twentieth century as the idea that they are due to pathological (‘irritative’) neuronal activity in the auditory cortex^6^. A current version of this approach proposes that, in addition to there being such a ‘bottom-up’ abnormal perceptual process, ‘top-down’ influences on perception act to confer additional features on AVH, leading them to be interpreted as the voices of family, friends or people involved in a conspiracy against the patient, etc^7^.

Complex perceptual experiences are known to occur in neurological disorders such as epilepsy and migraine, and electrical stimulation of the temporal lobe cortex in patients undergoing brain surgery can also result in auditory experiences up to and including speech^8^. Beyond such clinical observations, the neurological/perceptual model of AVH is testable using functional imaging, specifically the symptom capture paradigm, which compares brain activations at times when hallucinating patients hear a voice (which they typically signal by a button press) to periods when the voices are silent. Several studies of this type^9–12^ have found evidence of AVH-related activations in the superior temporal cortex, the posterior part of which contains the primary auditory cortex (Heschl’s gyrus). Others, however, have not found auditory cortex activation^13^, or found activation only in a very small cluster (11 voxels)^14^ or activation which appeared to be localized to temporal lobe white matter^15^.

We employed a novel variant of the symptom capture paradigm and 3 T fMRI to examine 30 right-handed adult patients with a DSM-5 diagnosis of schizophrenia or schizoaffective disorder (see “Methods” for diagnostic and exclusion criteria). Fifteen of these patients (the AVH + group) reported experiencing AVH nearly continually. The remaining 15 patients (the AVH- group) had been free of AVH for at least six months. The two groups were matched for age (*t* = 1.53, *p* = 0.14), sex (χ^2^ = 0.75, *p* = 0.39), and premorbid IQ, as estimated using a word pronunciation test (*t* = 0.25, *p* = 0.81) (see “Methods” and Supplementary Table S1).

**Figure 1:**
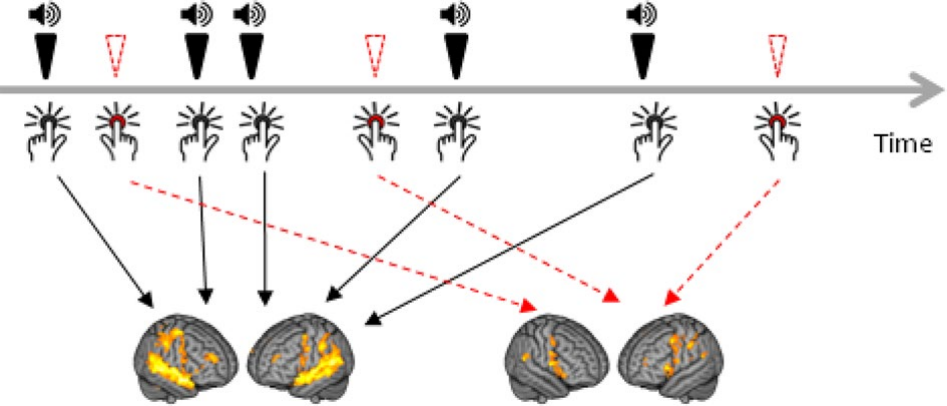
Overview of the experimental paradigm. Throughout the 10 min, 10 s scanning period, participants pressed a button with their right index finger when they experienced an auditory hallucination (red), and with their left index finger when real speech was presented through headphones (black).

During the functional run (lasting 10 min 10 s) the AVH + patients were instructed to press a button with their right index finger each time they heard a voice (frequency of button press during scanning ranged from 5 to 174 times, mean 43.53, SD = 49.20, median = 23). During the same run 40 randomly timed examples of real speech were also delivered to both ears via MRI-compatible headphones, to which the patients had to respond with the left index finger (see Fig. 1). The real speech was individually tailored to be similar in form to each patient’s AVH. To achieve this, prior to scanning the patients were asked to repeat out loud what their voices said, as they heard them, over a 5-min period, and their verbatim responses were tape-recorded and transcribed (for more details, see “Methods”). Examples, which took the form of single words, short phrases or sentences such as ‘The good boy’ or ‘You will change the world’, were then recorded for presentation during scanning in a neutral voice by an individual of the same gender of the hallucinated voice, as reported by each patient. The real speech stimuli were separated by random intervals ranging from 3 to 30 s (mean = 14.94 s, SD = 7.06); stimulus duration ranged from 0.53 to 3.22 s (mean = 1.33, SD = 0.67).

The AVH− patients performed the second part of the fMRI task only. The real speech stimuli presented to these patients were the same as the ones presented to AVH + patients: for each patient, the stimuli that were used were taken from an AVH + patient who was as similar as possible in terms of age, sex and estimated premorbid IQ.

## Results

### Activations in response to AVH and real auditory stimuli

**Figure 2:**
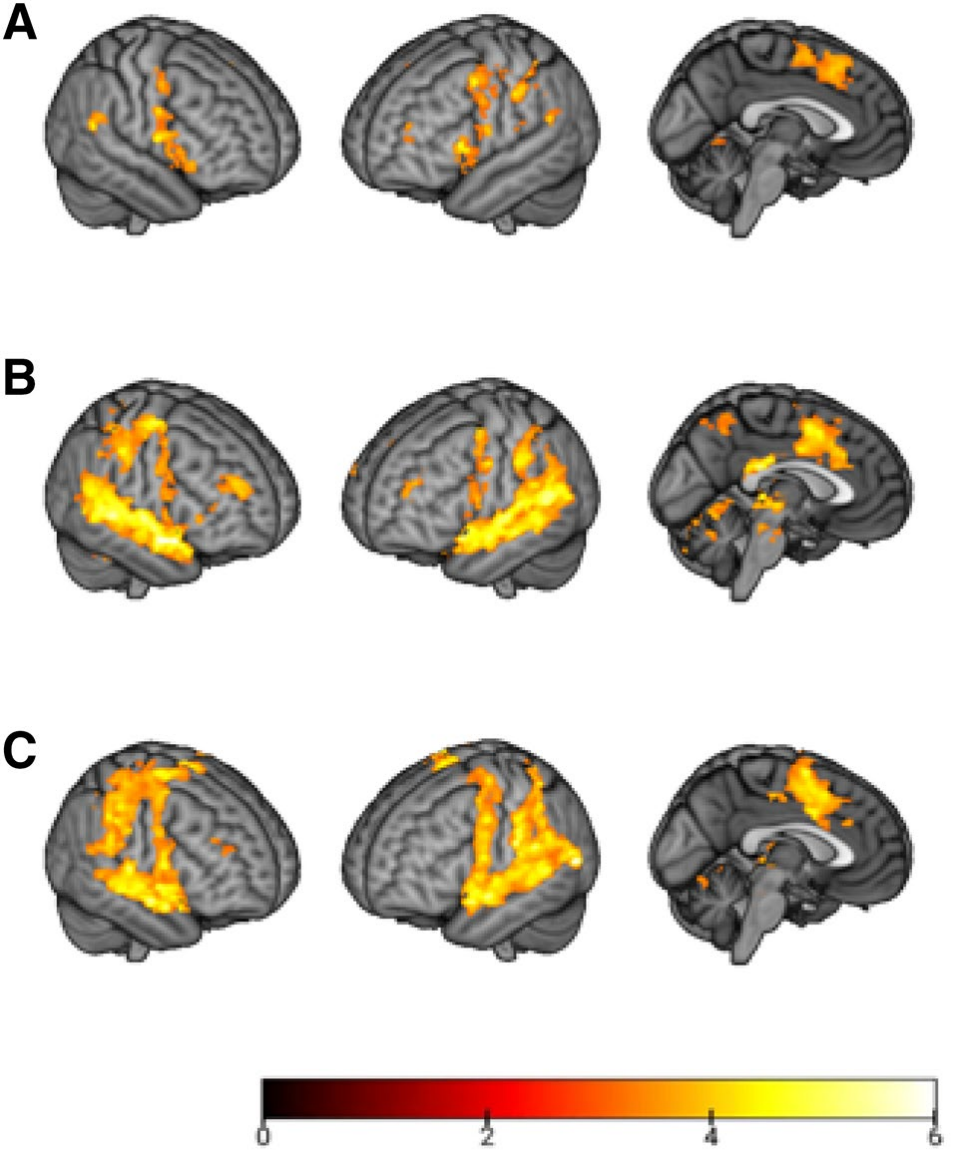
Group activation maps for experience of auditory hallucinations **(A)** and real speech **(B)** in 15 hallucinating patients. Activations to real speech in 15 non-hallucinating patients are shown in row **(C)**. Colour bar depicts *Z* values.

Findings using whole brain, voxel-based analyses, with an initial threshold of *z* = 3.1 (*p* < 0.001) and cluster-corrected for multiple comparisons at *p* < 0.05, are shown in Fig. 2 (for full details of the data analysis see “Methods”; MNI coordinates for all clusters are given in Supplementary Table S2). As a group, the AVH + patients showed no activation in most of the superior temporal cortex when they experienced AVH, including its posterior portion which contains the primary auditory cortex (Heschl’s gyrus). The only exception was a bilateral cluster in the extreme posterior superior temporal gyrus and the adjacent supramarginal gyrus, which on the left includes the regions usually identified as Wernicke’s area.

Experience of AVH was, however, associated with activations in circumscribed regions outside the temporal lobe. As well as Wernicke’s area and its right homologue, these included the bilateral inferior frontal gyrus (Broca’s area and its right homologue), the precentral gyrus and the supplementary motor area, both bilaterally.

In contrast, when hearing real speech, the AVH + patients showed activation along the length of the superior temporal cortex bilaterally, as well as in areas outside this (see Fig. 2, panel B). The extra-temporal areas activated largely overlapped with, but were more extensive than, the regions activated by experience of hallucinations. Activations in response to hearing real speech were closely similar in the AVH- patients (see Fig. 2, panel C and Supplementary Table S3).

### Region of interest analysis in hallucinators

**Figure 3:**
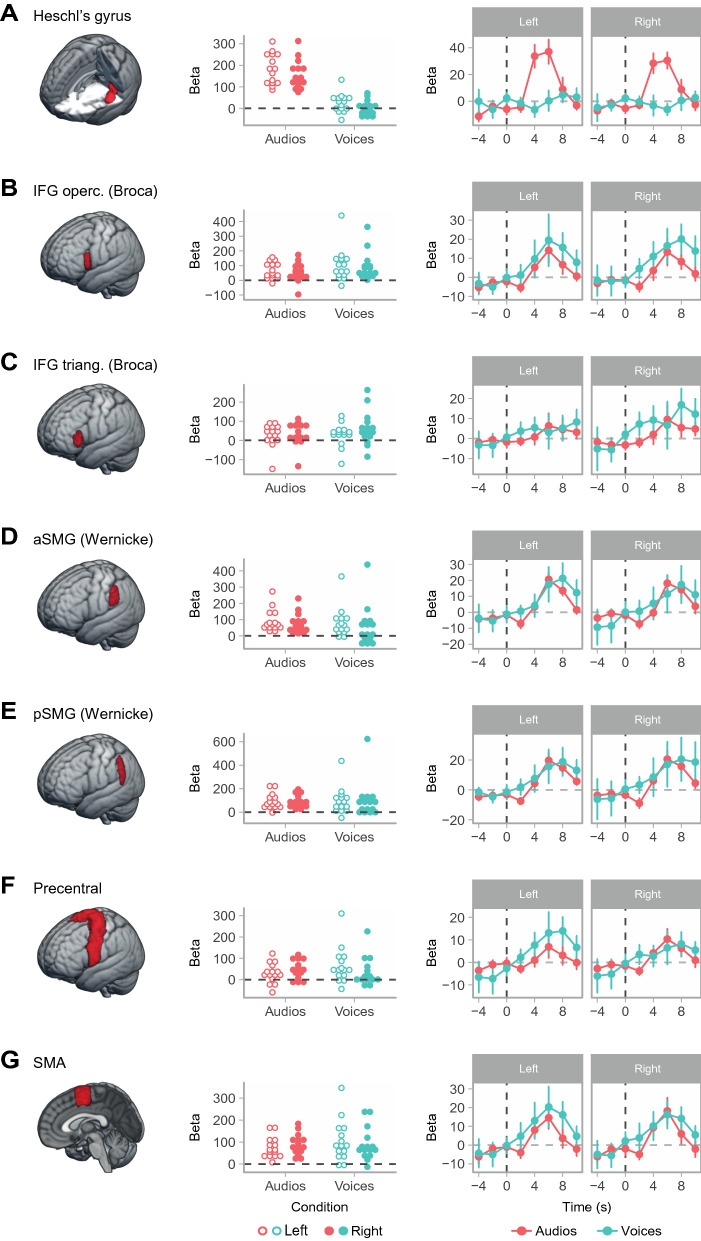
Task-related activation in anatomically-defined ROIs for auditory perception, language processing and motor regions. Dot plots show individual mean activation levels (beta values) for real auditory stimuli and AVH in Heschl’s gyrus **(A)**, the inferior frontal gyrus, pars opercularis **(B)**, the inferior frontal gyrus, pars triangularis **(C)**, the anterior portion of the supramarginal gyrus **(D)**, the posterior supramarginal gyrus **(E)**, the precentral gyrus **(F)** and the supplementary motor area **(G)**. Time-series plots show group signal change in the same ROIs relative to the region’s average, in the time window spanning 4 s before to 10 s after stimulus presentation (estimated stimulus occurrence time in the case of AVH).

We further explored the AVH + patients’ responses to hallucinated and real speech using time series plots of activation in anatomically-defined regions of interest (ROIs) (using the Harvard–Oxford Cortical Structural Atlas: https://fsl.fmrib.ox.ac.uk/fsl/fslwiki/Atlases) in regions where activation was found in the two conditions. As shown in Fig. 3, Heschl’s gyrus was robustly activated in response to real speech, but activation barely rose above baseline in response to AVH. In contrast, activation levels for both real speech and AVH were similar in the two regions generally accepted as comprising Broca’s area, the left inferior frontal gyrus, pars opercularis and pars triangularis, and in its homologue on the right. This was also the case for the anterior and posterior portions of the supramarginal gyrus, which on the left overlap with Wernicke’s area^16^. Finally, activations were similar for AVH and real speech in the precentral gyrus and supplementary motor area; these activations may have reflected the effect of button-pressing, but the ventral premotor cortex has also been suggested to play a role in speech perception^17–19^.

### Examination of possible interfering effects of real auditory stimuli on activations to AVH

Given that the design of the study meant that AVH occurred in the same blocks as the presentation of real auditory stimuli, it needs to be considered whether auditory cortex activations produced by real speech might have obscured activations to AVH occurring very soon afterwards. As a first test of this, we measured the correlation between the regressors for AVH and real speech in the individual GLMs in our first-level model. In all cases, this was close to zero or negative (mean = − 0.14, range =  + 0.04 to − 0.48). This finding does not suggest that temporal co-occurrence between AVH and the real speech stimuli was playing an important role.

Secondly, we ran an additional analysis to minimize any interfering effects of auditory stimuli on AVH-related activations occurring very shortly afterwards. At the first level, we defined three regressors: real auditory stimuli, hallucinations occurring more than 10 s after a preceding auditory stimulus, and hallucinations occurring up to 10 s after a preceding auditory stimulus (a 10-s separation was chosen based on the time-series plots shown in Fig. 3, which indicate that activation linked to real auditory stimuli had returned to baseline levels after this interval). The last regressor was considered a nuisance regressor. Motion regressors were also included as covariates, as in the original model. At the second level, we examined activations to AVH occurring more than 10 s after a preceding auditory stimulus with a one-sample t-test (p < 0.05, cluster-corrected, with a cluster-defining threshold of z > 3.1).

The findings are shown in Supplementary Figure S1 and Supplementary Table S4; the pattern of activation remained similar, although with smaller cluster sizes reflecting the smaller number of events captured, and the auditory cortex continued to be uninvolved. As in the original analysis, we found a small cluster of activation in the extreme posterior superior temporal gyrus, in this case only in the right hemisphere, roughly overlapping with Wernicke’s area right homologue.

## Discussion

Contradicting several earlier studies using the symptom capture paradigm^10–12^ as well as meta-analyses of such studies^20–22^, we found nothing to suggest that the experience of AVH is associated with auditory cortex activation. This failure, coupled with the fact that perception of formally similar real speech strongly activated a large expanse of the superior temporal cortex, would seem to exclude theoretical approaches to AVH in schizophrenia that invoke abnormal neuronal activity in the auditory cortex.

This then implies that some version of the cognitive model of AVH must be correct. It should be noted that the findings of our study make one theory of this type—that AVH are misinterpreted vivid/intrusive memories—unlikely. The brain functional correlates of the conscious re-experiencing of memories, or autobiographical recall, are well established^23^ and prominent among them are activations in two midline cortical regions, the medial frontal cortex and the posterior cingulate gyrus/precuneus. These two areas are a key part of the default mode network, a set of brain regions that typically de-activate during the performance of most cognitive tasks^24^; autobiographical recall is one of a small number of tasks that have been found to activate rather than de-activate this network^25^. There was no hint of a default mode network pattern of activation in response to AVH in our study. Nor have meta-analyses of symptom capture studies suggested such an activation pattern^20–22^.

We did find activations outside the temporal lobe during experience of AVH: these involved Wernicke’s area and its right homologue, Broca’s area and its right homologue, and the precentral gyrus and supplementary motor area bilaterally. While, as noted above, activations in motor areas may simply have reflected the act of button-pressing, our finding of activations in Broca’s and Wernicke’s areas (and perhaps also the activations in the ventral premotor cortex, which has been considered to play a role in speech perception^17–19^) could be taken to suggest that the experience of AVH involves mechanisms that normally participate in the processing of speech. Given the lack of accompanying auditory cortex activation, this would presumably be at the level of decoding of the linguistic properties of speech rather than its initial detection and analysis of its auditory perceptual qualities.

There is also another possible interpretation of the pattern of AVH-related activations we found. Based on functional imaging studies^26–30^, Broca’s area, the precentral cortex and the supplementary motor area are regarded as core regions subserving working memory, specifically its verbal non-executive component, the articulatory or phonological loop. Interestingly, a further region is implicated in verbal short-term memory, the left supramarginal gyrus, which has been argued to fulfil the temporary storage or ‘buffering’ function of the articulatory/phonological loop^31^. This region was also activated in our study, as part of the cluster of activation in Wernicke’s area (which includes the supramarginal gyrus according to current views^16^). Since verbal short-term memory equates to some extent with the concept of inner speech^32^, our findings could therefore be interpreted as providing support for Frith’s^3^ mislabelled inner speech theory of AVH.

Our findings pertain to AVH as experienced by patients with schizophrenia. However, it is now well documented that around 6% of healthy adults also report having experienced AVH^33^. To date, two symptom capture studies have examined the functional imaging correlates of AVH in such ‘healthy voice hearers’. Using whole brain analysis in seven AVH-experiencing individuals, Linden et al^34^ found a pattern of activations that was not dissimilar to the one we found, in that it included Broca’s and Wernicke’s areas and their right homologues; however, regions of the prefrontal, parietal and temporal lobe cortex were also activated. Interestingly, the temporal lobe activation did not appear to involve the primary auditory cortex but rather the superior temporal sulcus and the planum temporale, which lies immediately posterior to Heschl’s gyrus. Rather differently, Diederen et al^35^ examined 21 healthy voice hearers and 21 matched hallucinating patients with schizophrenia or other psychotic disorders. Examination of ROIs previously reported to be involved in the experience of AVH revealed evidence of bilateral superior temporal gyrus activation in the two groups combined, with no differences between the psychotic and healthy individuals in a conjunction analysis.

Some limitations of the study need to be acknowledged. Like most functional imaging studies of schizophrenia, it was carried out on patients who were receiving antipsychotic drug treatment. Also, the design of the study meant that activations attributable to the act of button pressing could not be separated from those due to experience of AVH. A final potential confound arises from the design of the study, which measured activations to both AVH and real auditory stimuli in the same scanning session, and is a departure from previous studies. This design meant that any particularly long-lasting AVH would have had the capacity to affect and interfere with the baseline. While we took precautions to avoid this possibility, using a regressor for AVH events in the first-level GLM (2.33 s) that was longer than the mean duration of AVHs based on the pre-scanning interview (1.33 s), this might not have been sufficient to avoid such interfering effects all of the time. Further studies examining AVH and real auditory stimuli in different blocks would accordingly be desirable to confirm the present study’s findings.

## Methods

### Participants

The final samples of AVH + and AVH− patients were drawn from a larger sample of patients with schizophrenia/schizoaffective disorder (see Supplementary Information for reasons for exclusion of patients from the two groups). They were recruited from four different hospitals in the metropolitan area of Barcelona, Spain (Benito Menni CASM, Hospital de Sant Rafael, Hospital Sagrat Cor de Martorell, Hospital Mare de Déu de la Mercè). Diagnosis was made by means of structured psychiatric interview and review of case notes. Patients were excluded if they (a) were younger than 18 or older than 65 years, (b) had a history of brain trauma or neurological disease, and (c) had shown alcohol/substance abuse within 12 months prior to participation. We included one patient with daily cannabis use who did not show evidence of abuse/dependence. Patients with an estimated premorbid IQ and/or current IQ (see below) < 70 were excluded from the study. All patients were taking antipsychotic medication.

All participants gave written informed consent prior to participation. All the study procedures complied with the ethical standards of the relevant national and institutional committees on human experimentation and with the Helsinki Declaration of 1975, as revised in 2008. The study was approved by the local hospital ethics committee (Comité Ético de Investigación Clínica, CEIC), of Hospital Benito Menni, which has responsibility for all the hospitals involved. The participants did not receive any economic compensation.

### Clinical assessment

AVH severity was assessed with the Psychotic Symptom Rating Scale auditory hallucinations subscale (PSYRATS-H)^36^. This subscale consists of a semi-structured interview with 11 items referring to frequency, duration, controllability, loudness, location severity and intensity of distress; amount and degree of negative content; beliefs about the origin of voices; and disruption caused by the AVH. Premorbid IQ was estimated using the Word Accentuation Test (Test de Acentuación de Palabras, TAP^37,38^): this requires pronunciation of low-frequency Spanish words whose accents have been removed. Current IQ was estimated using four subtests from the WAIS-III (Vocabulary, Similarities, Matrix reasoning and Block Design). All assessments took place within 1 week of the scanning session.

### Task and stimuli

Prior to the scanning session, participants experiencing AVH were interviewed to obtain samples of their AVH content. In a quiet environment over a period of five minutes, they were asked, whenever they experienced an instance of AVH, to repeat its content out loud. What they said was tape-recorded and transcribed by the interviewer. Additional samples were obtained by asking the participants to report the content of AVH experienced earlier that day or in the past days. Qualitative features such as tone, or the voice being male or female, were also registered. After each interview, five of the provided examples were randomly selected and recorded either in a male or female voice, as indicated by the participant, to be presented as individually tailored auditory stimuli during the symptom capture task. Stimuli were recorded and normalized to a 60-dB intensity with the Praat software (http://www.fon.hum.uva.nl/praat/), which was also used to remove silence periods at the beginning and end of each recording.

During scanning, auditory stimuli were presented via MRI-compatible headphones. To keep visual stimulation similar for all patients, the task was performed with eyes open, while viewing a grey screen via MRI-compatible goggles (VisuaStim, Resonance Technology, Northridge, CA, USA). Responses were registered with MRI-compatible response grips (NordicNeuroLab, Bergen, Norway).

### Image acquisition and analysis

Images were acquired with a 3 T Philips Ingenia scanner (Philips Medical Systems, Best, The Netherlands). Functional data were acquired using a T2*-weighted echo-planar imaging (EPI) sequence with 310 volumes and the following acquisition parameters: TR = 2000 ms, TE = 30 ms, flip angle = 70°, in-plane resolution = 3.5 × 3.5 mm, FOV = 238 × 245 mm, slice thickness = 3.5 mm, inter-slice gap = 0.75 mm. Slices (32 per volume) were acquired with an interleaved order parallel to the AC-PC plane. We also acquired a high-resolution anatomical volume with a FFE (Fast Field Echo) sequence for anatomical reference and inspection (TR = 9.90 ms; TE = 4.60 ms; Flip angle = 8°; voxel size = 1 × 1 mm; slice thickness = 1 mm; slice number = 180; FOV = 240 mm).

Preprocessing and analysis were carried out with the FEAT module included in the FSL (FMRIB Software Library) software^39^. The first 10 s (5 volumes) of the sequence, corresponding to signal stabilization, were discarded. Preprocessing included motion correction (using the MCFLIRT algorithm), co-registration, and normalization to a common stereotactic space (MNI, Montreal Neurological Institute template). For accurate registration, a two-step process was used. First, brain extraction was applied to the structural image, and the functional sequence was registered to it. Then the structural image was registered to the standard template. These two transformations were used to finally register the functional sequence to the standard space. Before group analyses, normalized images were spatially filtered with a Gaussian filter (FWHM = 5 mm). To minimize unwanted movement-related effects, individuals with an estimated maximum absolute movement > 3.0 mm or an average absolute movement > 0.3 mm were excluded from the study.

Statistical analysis was performed by means of a General Linear Model (GLM) approach. A first regressor was defined with onset times for auditory stimuli, with duration set, for each subject, as their mean reaction time (RT, calculated as time elapsed between stimulus onset and button press) plus 2 SDs. This duration was set to encompass the presentation of the auditory stimulus and the motor response. Mean duration for this regressor was 2.33 s (SD = 0.94 s). For patients with AVH, we also defined a second regressor corresponding to AVH occurrence. AVH onset times were set by subtracting stimulus duration (mean RT + 2*SD) to button-press times signaling AVH occurrence, with the same duration as the auditory stimuli. Responses made less than 1 s apart were considered to belong to the same AVH instance. Motion parameters obtained from realignment were also included as nuisance covariates.

GLMs were fitted to generate individual activation maps for auditory stimuli and for AVH occurrence (the last only in the AVH-experiencing group). Second level (group) analyses were performed within the FEAT module by means of mixed-effects GLMs^40^, to obtain mean activation maps for each group with one-sample t-tests. All statistical tests were carried out at the cluster level with a corrected *p* < 0.05 using Gaussian random field methods, with a threshold of *z* = 3.1 (*p* < 0.001) to define the initial set of clusters.

## Supplementary Information

Supplementary Information.

## Data Availability

The data sets generated during and/or analysed during the current study are available from the corresponding author on reasonable request.
