# Supplementary material for: Auditory hallucinations activate language and verbal short-term memory, but not auditory, brain regions
Source: Sci Rep. 2021 Sep 23;11:18890. doi: 10.1038/s41598-021-98269-1 (PMC8460641; doi:10.1038/s41598-021-98269-1)
Supplement: Supplementary file 1 — Supplementary Information. [file 41598_2021_98269_MOESM1_ESM.docx]

**Supplementary Information**

**Excluded patients**

From a total of 64 patients screened, 49 completed the symptom capture task (24 in the AVH+ and 25 in the AVH- groups). From the AVH+ group, six patients were further excluded due to not experiencing any AVH during the task, one due to excessive head motion, and two for not following task instructions, leaving a final sample of 15 AVH+ patients. From the AVH- group, four patients were excluded for not following task instructions (i.e., responding at random moments or not responding when stimuli were presented) and two due to excessive head motion. From the remaining 19 AVH- patients, 15 were selected to provide the best match to the AVH+ group in terms of age, gender and IQ.

**Supplementary Results**

**Table S1.** Regions of activation in response to auditory hallucinations.

|  |  | **MNI coordinates** | | |  | **Cluster** |  |
| --- | --- | --- | --- | --- | --- | --- | --- |
| **Region** | **Hemisphere** | ***x*** | ***y*** | ***z*** | **Z** | **size** | ***p*** |
| Precentral gyrus | L | -42 | 4 | 50 | 4.91 | 2010 | <0.001 |
| Postcentral gyrus | L | -34 | -20 | 52 | 4.91 |  |  |
| Middle frontal cortex | L | -26 | -2 | 54 | 4.73 |  |  |
| Inferior parietal cortex | L | -40 | -28 | 40 | 4.28 |  |  |
| SMA | L | -10 | 12 | 58 | 5.1 | 1979 | <0.001 |
| SMA | R | 6 | 10 | 52 | 4.15 |  |  |
| IFG *pars triangularis* | L | -50 | 22 | 12 | 5.01 | 1325 | <0.001 |
| Temporal pole | L | -50 | 6 | 0 | 4.35 |  |  |
| Insula | L | -42 | 0 | 6 | 4.33 |  |  |
| IFG *pars orbitalis* | L | -50 | 20 | -2 | 4.27 |  |  |
| Precentral | R | 58 | 8 | 18 | 4.91 | 889 | <0.001 |
| IFG *pars opercularis* | R | 48 | 12 | 4 | 4.16 |  |  |
| Insula | R | 36 | 24 | 4 | 4.07 |  |  |
| Rolandic operculum | R | 54 | 8 | 10 | 4.02 |  |  |
| Cerebellum | R | 14 | -72 | -24 | 4.2 | 733 | <0.001 |
| Cerebellum | L | -34 | -70 | -22 | 4.23 | 272 | <0.001 |
| Cerebellum | R | 10 | -50 | -10 | 3.87 | 202 | <0.001 |
| Precentral gyrus | R | 40 | -6 | 60 | 3.84 | 182 | <0.001 |
| Superior temporal gyrus | R | 68 | -40 | 22 | 4.63 | 149 | 0.002 |
| Middle frontal gyrus | L | -30 | 48 | 24 | 3.8 | 129 | 0.006 |
| Superior temporal gyrus | L | -60 | -42 | 24 | 4.38 | 126 | 0.007 |

SMA: supplementary motor area; IFG: inferior frontal gyrus; L: left; R: right.

**Table S2.** Regions of activation in response to auditory stimuli in the hallucinating group.

|  |  | **MNI coordinates** | | |  | **Cluster** |  |
| --- | --- | --- | --- | --- | --- | --- | --- |
| **Region** | **Hemisphere** | ***x*** | ***y*** | ***z*** | **Z** | **size** | ***p*** |
| Temporal pole | R | 54 | 8 | -12 | 6.59 | 27194 | <0.001 |
| Superior temporal gyrus | R | 52 | -18 | -2 | 6.41 |  |  |
| Superior temporal gyrus | L | -50 | -4 | -8 | 6.26 |  |  |
| Heschl’s gyrus | L | -38 | -26 | 8 | 6.09 |  |  |
| Middle temporal gyrus | L | -48 | -56 | 12 | 5.75 |  |  |
| Inferior parietal cortex | L | -34 | -50 | 44 | 5.42 |  |  |
| IFG *pars orbitalis* | L | -46 | 18 | -10 | 5.33 |  |  |
| Cerebellum | L | -30 | -50 | -30 | 4.95 | 2665 | <0.001 |
|  | R | 16 | -70 | -24 | 4.66 |  |  |
| Midbrain | L | -8 | -30 | -2 | 4.87 | 1483 | <0.001 |
|  | R | 6 | -32 | -4 | 4.72 |  |  |
| Middle frontal gyrus | R | 26 | 50 | 18 | 4.84 | 617 | <0.001 |
| Precuneus | L | -12 | -54 | 44 | 4.75 | 566 | <0.001 |
|  | R | 8 | -62 | 44 | 3.90 |  |  |
| Posterior cingulate | L | -2 | -32 | 24 | 5.25 | 426 | <0.001 |
|  | R | 6 | -38 | 24 | 4.53 |  |  |
| Middle frontal gyrus | L | -30 | 42 | 26 | 4.62 | 370 | <0.001 |

SMA: supplementary motor area; IFG: Inferior frontal gyrus; L: left; R: right.

**Table S3.** Regions of activation in response to auditory stimuli in the non-hallucinating group.

|  |  | **MNI coordinates** | | |  | **Cluster** |  |
| --- | --- | --- | --- | --- | --- | --- | --- |
| **Region** | **Hemisphere** | ***x*** | ***y*** | ***z*** | **Z** | **size** | ***p*** |
| Superior temporal gyrus | R | 58 | 0 | -10 | 6.54 | 26552 | <0.001 |
| Postcentral gyrus | L | -60 | -18 | 28 | 6.08 |  |  |
| Temporal pole | R | 54 | 10 | -14 | 5.82 |  |  |
| Supramarginal gyrus | L | -56 | -22 | 34 | 5.79 |  |  |
| Superior temporal gyrus | L | -60 | -32 | 24 | 5.55 |  |  |
| Inferior parietal cortex | L | -46 | -38 | 42 | 5.52 |  |  |
| Supramarginal gyrus | R | 48 | -32 | 42 | 5.52 |  |  |
| Temporal pole | L | -50 | 12 | -12 | 5.31 |  |  |
| Inferior parietal cortex | R | 52 | -34 | 50 | 5.22 |  |  |
| SMA | L | -10 | 0 | 52 | 5.12 |  |  |
| SMA | R | 4 | -6 | 74 | 5.11 |  |  |
| Insula | L | -40 | -2 | 6 | 5.06 |  |  |
| Cerebellum | L | -42 | -60 | -32 | 4.14 | 879 | <0.001 |
|  | R | 2 | -68 | -14 | 3.95 |  |  |
| Cerebellum | R | 28 | -58 | -26 | 5.16 | 637 | <0.001 |
| Thalamus | L | -8 | -20 | 6 | 4.55 | 526 | <0.001 |
| Midbrain | L | -14 | -26 | -4 | 4.3 |  |  |
| Cerebellum | R | 18 | -72 | -48 | 5.17 | 211 | <0.001 |
| Middle frontal gyrus | R | 28 | 46 | 24 | 4.63 | 191 | <0.001 |

SMA: supplementary motor area; IFG: inferior frontal gyrus; L: left; R: right.

**Table S4.** Regions of activation in response to auditory hallucinations, limited to hallucination events more than 10 seconds apart from auditory stimuli.

|  |  | **MNI coordinates** | | |  | **Cluster** |  |
| --- | --- | --- | --- | --- | --- | --- | --- |
| **Region** | **Hemisphere** | ***x*** | ***y*** | ***z*** | **Z** | **size** | ***p*** |
| SMA | B | -2 | 18 | 44 | 4.76 | 1684 | <0.001 |
| Insula/IFG | L | -30 | 30 | 2 | 4.5 | 1012 | <0.001 |
| Rolandic operculum | L | -48 | 8 | 2 | 4.47 |  |  |
| IFG *pars triangularis* | L | -50 | 22 | 12 | 4.29 |  |  |
| Inferior parietal cortex | L | -46 | -28 | 40 | 4.17 | 626 | <0.001 |
| Postcentral gyrus | L | -56 | -20 | 52 | 4.08 |  |  |
| Precentral gyrus | R | 58 | 8 | 18 | 4.63 | 623 | <0.001 |
| Insula | R | 34 | 28 | 2 | 4.35 |  |  |
| Rolandic operculum | R | 50 | 10 | 4 | 4.18 |  |  |
| IFG *pars opercularis* | R | 56 | 20 | 10 | 4.04 |  |  |
| IFG *pars triangularis* | R | 50 | 24 | 0 | 3.96 |  |  |
| Precentral gyrus | L | -30 | -4 | 58 | 4.43 | 583 | <0.001 |
| Postcentral gyrus | L | -34 | -22 | 52 | 4.26 |  |  |
| Middle frontal gyrus | L | -42 | 4 | 52 | 3.97 |  |  |
| Cerebellum | R | 32 | -56 | -32 | 4.11 | 358 | <0.001 |
| Superior temporal gyrus | R | 68 | -40 | 22 | 4.12 | 104 | 0.0114 |
| Precentral gyrus | R | 58 | 8 | 34 | 3.96 | 83 | 0.0349 |
| Middle frontal gyrus | L | -30 | 52 | 18 | 3.63 | 83 | 0.0349 |

SMA: supplementary motor area; IFG: inferior frontal gyrus; L: left; R: right, B: both.

**Supplementary Figure 1.** Group activation map for auditory hallucinations occurring more than 10 seconds apart from auditory stimulation. Color bar depicts *z* values.

**
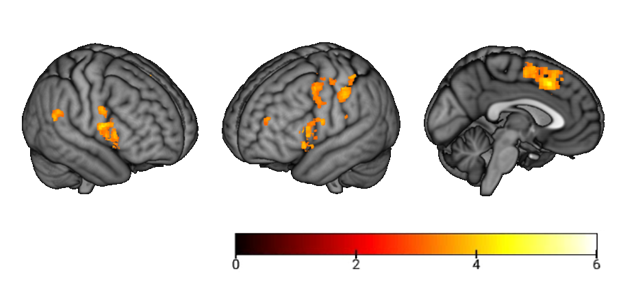
**
